# Supplementary material for: Analysis of Arbuscular Mycorrhizal Fungal Inoculant Benchmarks
Source: Microorganisms. 2020 Dec 31;9(1):81. doi: 10.3390/microorganisms9010081 (PMC7824734; doi:10.3390/microorganisms9010081)
Supplement: Supplementary file 1 [file microorganisms-09-00081-s001.zip › Supplementary Materials-microorganisms-1033676-final version-received from author 2020.12.31/Supplementary Materials-2020.12.31.pdf]

Supplementary Materials

Figure S1: Physical forms of the commercial mycorrhizal inoculants,

Figure S2: Breakdown of the commercial mycorrhizal inoculants by species composition (A); number of species per product (B); and active ingredients contained in formulations (C).

Figure S3: Each circle represents a different product and is positioned according to 3 characteristics: total mycorrhiza concentration in the product (propagules per gram), forms (liquid, powder or granular) and number of different mycorrhiza strains contained. Product names were not shown due to the space limitation.

Figure S4: Evolution of mycorrhizal producing and marketing firm in Europe from 2010 to 2017.

Table S1: Raw dada of AMF-based inoculants used in the study.

Table S2: Effect of Commercial Inoculants in Greenhouse and Field trials.

Supporting Figure S1

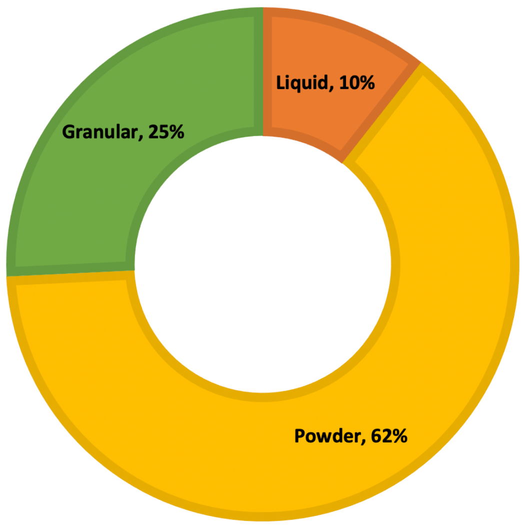

Supporting Figure S1. Physical forms of the commercial mycorrhizal inoculants.

Supporting Figure S2

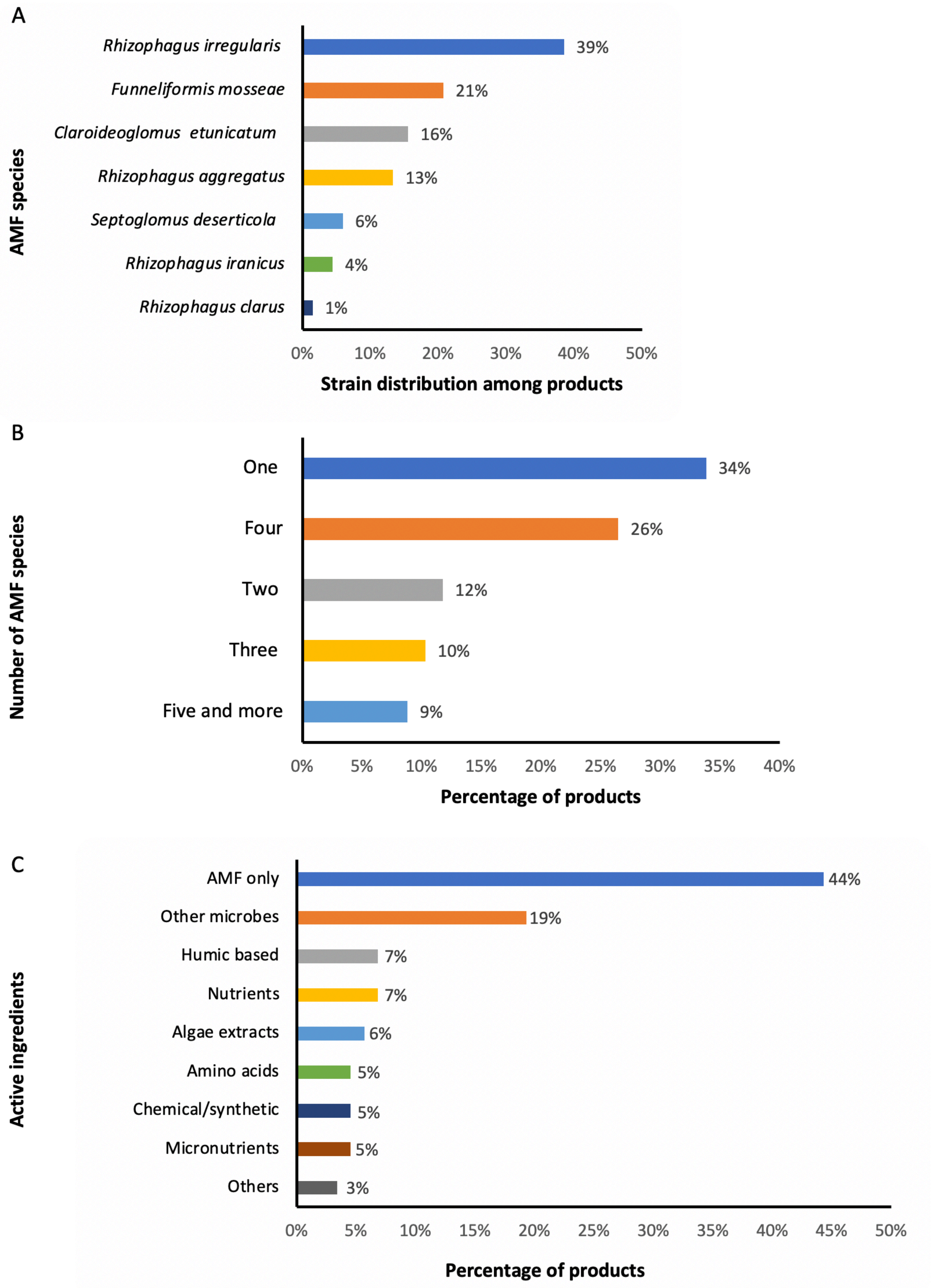

Supporting Figure S2. Breakdown of the commercial mycorrhizal inoculants by species composition (A); number of species per product (B); and active ingredients contained in formulations (C).

Supporting Figure 3

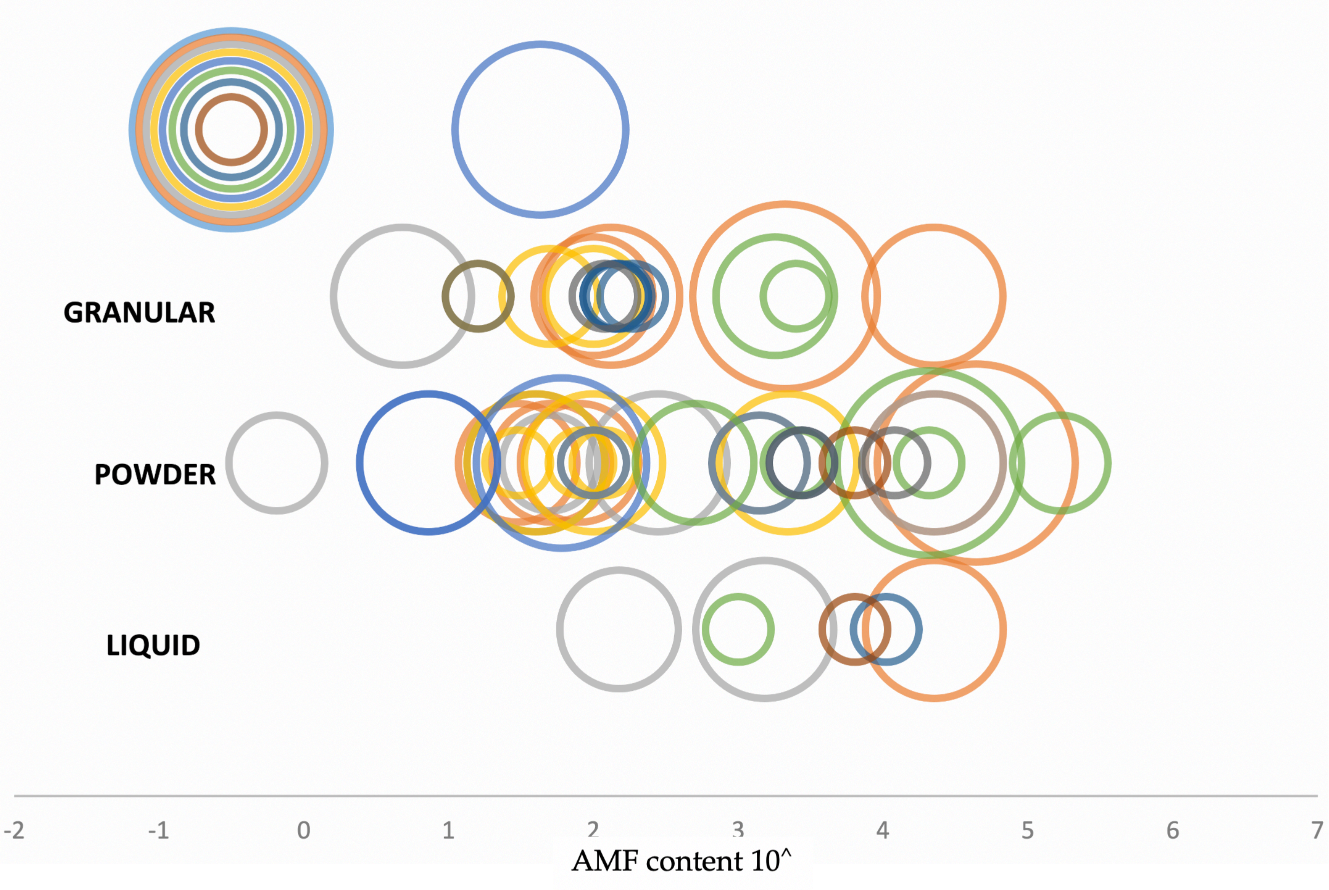

Supporting Figure S3. Each circle represents a different product and is positioned according to 3 characteristics: total mycorrhiza concentration in the product (propagules per gram), forms (liquid, powder or granular) and number of different mycorrhiza strains contained. Product names were not shown due to the space limitation.

Supporting Figure 4

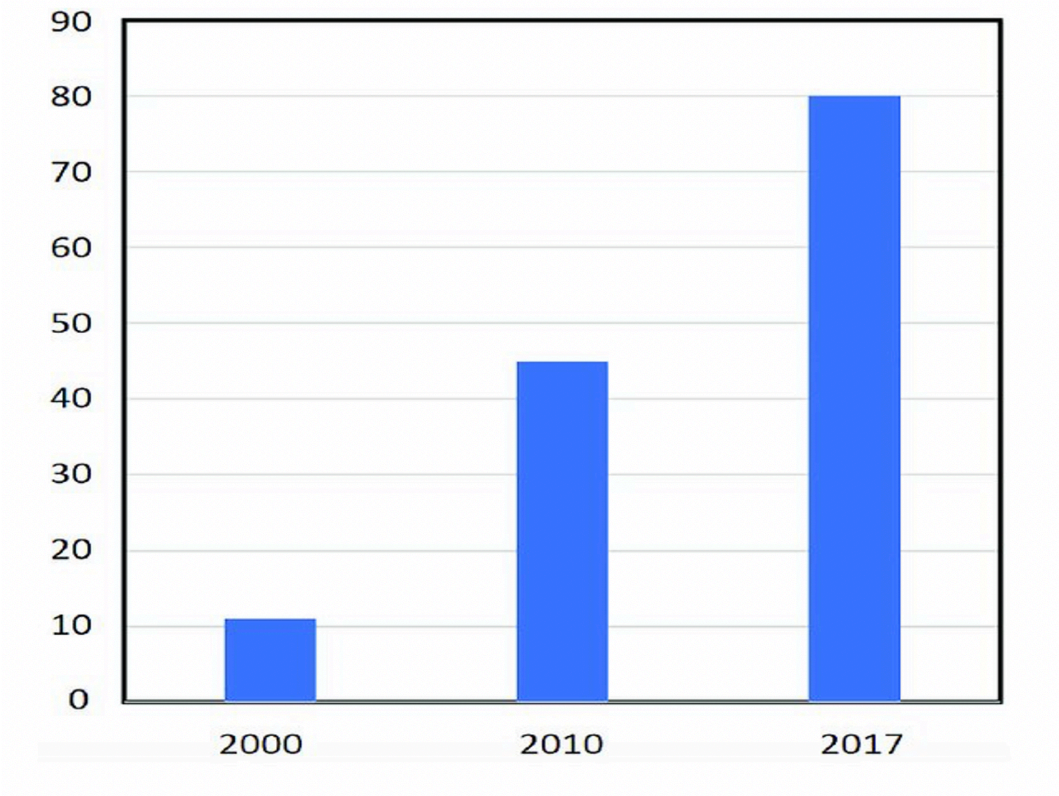

Supporting Figure S4. Evolution of mycorrhizal producing and marketing firm in Europe from 2010 to 2017. Adapted from Keswani et al., 2018.

Supporting Table S2: Effect of Commercial Inoculants in Greenhouse and Field trials.

Commercial inoculants	*AMF species*	Study conditions, country	Dosage applied	Crops	Effect of inoculants	Ref.
Myco Apply Soluble Endo MYCORRHIZAL APPLICATIONS, INC. (USA)	*G. intraradices, G. mosseae, G. aggregatum and G. etunicatum, G. intraradices, G. mosseae, G. aggregatum, G. monosporum, G. cralum, G. deserticola, Gi. margarita, Gi. brasilianum,*	Greenhouse Kenya	Not specified	Soybean	Increased root colonization	[1]
Myco Apply Endo plus MYCORRHIZAL APPLICATIONS, INC. (USA)	*G. intraradices, G. mosseae, G. aggregatum and G. etunicatum,Trichoderma konigii and T. harzianum*<br>*G. intraradices, G. mosseae, G. aggregatum, G. etunicatum, Rhizopogon villosullus, R. luteolus, R. amyopogon, R. fulvigleba,*	Greenhouse Kenya	Not specified	Soybean	Increased root colonization	[1]
Myco Apply e Endo MYCORRHIZAL APPLICATIONS, INC. (USA)	*G. intraradices, G. mosseae, G. aggregatum and G. etunicatum*	Greenhouse Kenya	Not specified	Soybean	Increased root colonization	[1]
Myco Apply Root Dip Gel MYCORRHIZAL APPLICATIONS, INC. (USA)	*Gi. etunicatum, R. vilosullus, R. lutelolus, R. amylopogon, R. fulvigleba, Pisolithus tinctorius, Scleroderma Cepa, S. cirtrinum propagules*	Greenhouse Kenya	Not specified	Soybean	Increased root colonization	[1]
Endorhize standard, AGRAUXINE (France)	*Glomus spp.*	Greenhouse Kenya	Not specified	Soybean	Increased root colonization	[1]
Endorhize premium AGRAUXINE (France)	*Glomus spp.*	Greenhouse and Field Kenya	Not specified	Soybean	Increased root colonization in the Greenhouse<br>Enhanced nodulation in the field<br>No significant yield increase in the field	[1]
Mycor IFTECH (France)	*Glomus intraradices*	Greenhouse Kenya	Not specified	Soybean	Increased root colonization	[1]
Vam-Tech NUTRI-TECH SOLUTIONS P/L (Australia)	*Glomus intraradices*	Greenhouse Kenya	Not specified	Soybean	Increased root colonization	[1]
Myco Apply Endo MYCORRHIZAL APPLICATIONS, INC. (USA)	*G. intraradices, G. mosseae, G. aggregatum and G. Etunicatum*	Greenhouse Kenya	Not specified	Soybean	Increased root colonization	[1]

Rhizatech<br>DUDUTECH (K) LTD.<br>(Kenya)	*Spores and mycelial fragments of AMF (mainly G. intraradices)*	Greenhouse and Field<br>Kenya	Not specified	Soybean	Increased root colonization in the Greenhouse<br>Enhanced nodulation in the field<br>No significant yield increase in the field	[1]
Zander Mycorrhiza<br>ZANDER MIDDLE EAST LLC, (United Arab Emirates)	Beneficial arbuscular mycorrhizal fungi from arid zones	Greenhouse<br>Kenya	Not specified	Soybean	Increased root colonization	[1]
MycoUp, MycoUp Activ, Resid HC and Resid MG<br>SYMBORG INC.[2]	*Glomus iranicum var. tenuihypharum*	Greenhouse and Field<br>Spain	3 kg/ha	Lettuce and grape	Increased biomass for lettuce under greenhouse,<br>improved fruit cluster weight, color uniformity and brix in grape in the field	[3]
MYKE® PRO Potato-L<br>PREMIER TECH BIOTECHNOLOGIES<br>(Canada)	*Glomus intraradices DAOM 197198*	Field	238 ml/ha	Irish Potato	Increased root colonization and yield	[4]
MYKE® PRO SG2<br>PREMIER TECH BIOTECHNOLOGIES<br>(Canada)	*Glomus intraradices*	Greenhouse<br>Canada	16 g/3L pot	Maize	Increased root colonization and yield under disturbed soil	[5]
MYKE® PRO GR<br>PREMIER TECH INC.<br>(Canada)	*Glomus intraradices*	Green house and Field<br>Canada	15g/7.6L pot	Grapevine Rootstocks	Increased biomass production in Greenhouse<br>Increased root colonization but no effect on biomass in the field	[6]
AEGIS®<br>ITALPOLLINA (Italy)	*Glomus intraradices*	Field<br>Italy	25 kg ha	Maize	Increased root colonization, yield and leaf biomass	[7]
Rootella BR<br>GROUNDWORK BIOAG<br>(Israel)	*Glomus intraradices*	Field<br>Brazil	1 kg/ha	Maize	Increased corn biomass and grain yield under varying P concentration	[8]
Product name not specified by author<br>SYMBIOM<br>(Czech republic)	*Claroideoglomus sp., Funneliformis sp., Diversispora sp., Glomus sp. and Rhizophagus sp.*	Greenhouse<br>China	10g/pot	Cucumber	Increased root colonization, increased plant height and dry weight, nutrient composition micro and macro	[9]
Product name not specified by author<br>SYMBIOM (Czech Republic)	*G. intradices, G. microageregatum BEG and G. Claroideum BEG 210*	Greenhouse<br>China	10g/pot	Cucumber	Increased root colonization, plant height and dry weigh, mineral composition	[9]

Micosat F<br>CCS S.R.L. AOSTA (Italy)	*Funneliformis coronatum GO01 and GU53, Funneliformis caledonium GM24, Rhizophagus intraradices GB67 and GG32, Funneliformis mosseae GP11 and GC11, and Septoglomus viscosum GC41), and three fungal saprotrophs (Beauveria spp. BB48, Trichoderma harzianum TH01, and Trichoderma atroviride TA28*	Field<br>Italy	10kg/ha	Maize	No inoculation, reduced dominance of local species, increased diversity of AMF community	[10]
Panoramix<br>Koppert Biological Systems, (The Netherlands),	*Consortium of AMF, PGPR, Bacillus sp. and Trichoderma*	Greenhouse<br>Italy	Seed coating, rate not available	*Dactylis glomerata L.* and mix of stand of grasses (*Lolium perenne L.*, *Poa pratensis L.* and *Festuca arundinacea Shreb*.).	Increased biomass, N uptake, reduce bioavailability of Cadmium, enhanced soil nitrogen fixing and ammonia and<br>oxidizing bacteria	[11]
Mycogrowth<br>SYMBORG	*Glomus iranicum var tenuihypharum*	Soilless growing system under Greenhouse	3kg/ha	Strawberry	Increased root colonization, and improve fruit quality by increasing content of anthocyanin and total phenolics	[12]

References.

1. Faye, A., et al., *Testing of commercial inoculants to enhance P Uptake and grain yield of promiscuous soybean in Kenya.* Sustainability, 2020. **12**(3803): p. 1-15.
2. Barker, S.J., et al., *A mutant in Lycopersicon esculentum Mill. with highly reduced VA mycorrhizal colonization: Isolation and preliminary characterisation.* Plant Journal, 1998. **15**(6): p. 791-797.
3. Félix Fernández, M., et al., *Application of Arbuscular Mycorrhizae Glomus iranicum var. tenuihypharum var. nova in Intensive Agriculture: A Study Case.* Journal of Agricultural Science and Technology B, 2017. **7**(4).
4. Hijri, M., *Analysis of a large dataset of mycorrhiza inoculation field trials on potato shows highly significant increases in yield.* Mycorrhiza, 2016. **26**(3): p. 209-14.
5. Antunes, P., et al., *Influence of commercial inoculation with Glomus intraradices on the structure and functioning of an AM fungal community from an agricultural site.* Plant and Soil, 2009. **317**: p. 257-266.
6. Rosa, D., et al., *Performance and Establishment of a Commercial Mycorrhizal Inoculant in Viticulture.* Agriculture, 2020. **10**(11).
7. Cozzolino, V., V. Di Meo, and A. Piccolo, *Impact of arbuscular mycorrhizal fungi applications on maize production and soil phosphorus availability.* Journal of Geochemical Exploration 2013. **129**: p. 40–44.
8. Stoffel, S.C.G., et al., *Yield increase of corn inoculated with a commercial arbuscular mycorrhizal inoculant in Brazil.* Ciência Rural, 2020. **50**(7).
9. Chen, S., et al., *Combined Inoculation with Multiple Arbuscular Mycorrhizal Fungi Improves Growth, Nutrient Uptake and Photosynthesis in Cucumber Seedlings.* Front Microbiol, 2017. **8**: p. 2516.
10. Berruti, A., E. Lumini, and V. Bianciotto, *AMF components from a microbial inoculum fail to colonize roots and lack soil persistence in an arable maize field.* Symbiosis, 2017. **72**: p. 73–80.
11. Visconti, D., et al., *Securing of an Industrial Soil Using Turfgrass Assisted by Biostimulants and Compost Amendment.* Agronomy, 2020. **10**(9).
12. Cecatto, A.P., et al., *Mycorrhizal inoculation affects the phytochemical content in strawberry fruits.* Acta Scientiarum. Agronomy, 2016. **38**(2).
